# Acetyl-dl-leucine in cerebellar ataxia ([^18^F]-FDG-PET study): how does a cerebellar disorder influence cortical sensorimotor networks?

**DOI:** 10.1007/s00415-022-11252-2

**Published:** 2022-07-25

**Authors:** Sandra Becker-Bense, Lena Kaiser, Regina Becker, Katharina Feil, Carolin Muth, Nathalie L. Albert, Marcus Unterrainer, Peter Bartenstein, Michael Strupp, Marianne Dieterich

**Affiliations:** 1 German Center for Vertigo and Balance Disorders (DSGZ), University Hospital, LMU Munich, Marchioninistr. 15, 81377 Munich, Germany; 2 Department of Nuclear Medicine, University Hospital, LMU Munich, Munich, Germany; 3 Department of Neurology, University Hospital, LMU Munich, Munich, Germany; 4 Department of Radiology, University Hospital, LMU Munich, Munich, Germany; 5 Munich Cluster of Systems Neurology (SyNergy), Munich, Germany

**Keywords:** Cerebellum, Ataxia, PET, Vestibular, Treatment, Acetyl-dl-leucine, Compensation

## Abstract

**Objective:**

The aim of the study was to deepen our insights into central compensatory processes of brain networks in patients with cerebellar ataxia (CA) before and with treatment with acetyl-dl-leucine (AL) by means of resting-state [^18^F]-FDG-PET brain imaging.

**Methods:**

Retrospective analyses of [^18^F]-FDG-PET data in 22 patients with CA (with vestibular and ocular motor disturbances) of different etiologies who were scanned before (PET A) and on AL treatment (PET B). Group subtraction analyses, e.g., for responders and non-responders, comparisons with healthy controls and correlation analyses of regional cerebral glucose metabolism (rCGM) with symptom duration, ataxia (SARA) and quality of life (QoL) scores were calculated.

**Results:**

Prior to treatment rCGM was consistently downregulated at the cerebellar level and increased in multisensory cortical areas, e.g., somatosensory, primary and secondary visual (including V5, precuneus), secondary vestibular (temporal gyrus, anterior insula), and premotor/supplementary motor areas. With AL (PET B vs. A) cerebellar hypometabolism was deepened and sensorimotor hypermetabolism increased only in responders with clinical benefit, but not for the non-responders and the whole CA group. A positive correlation of ataxia improvement with rCGM was found in visual and vestibular cortices, a negative correlation in cerebellar and brainstem areas. QoL showed a positive correlation with rCGM in the cerebellum and symptom duration in premotor and somatosensory areas.

**Conclusions:**

Central compensatory processes in CA mainly involve multisensory visual, vestibular, and somatosensory networks as well as premotor/primary motor areas at the cortical level. The enhanced divergence of cortical sensorimotor up- and cerebellar downregulation with AL in responders could reflect amplification of inhibitory cerebellar mechanisms.

**Supplementary Information:**

The online version contains supplementary material available at 10.1007/s00415-022-11252-2.

## Introduction

Cerebellar ataxia (CA) is a chronic defacing syndrome. Its common clinical features mainly encompass imbalance and incoordination due to gait and limb ataxia, impaired speech, as well as distinct ocular motor dysfunction, e.g., gaze-holding deficit, saccadic smooth pursuit or downbeat nystagmus [1, 2]. A number of heterogeneous innate and acquired disorders are included in the term “CA”. Degeneration typically involves the cerebellar parenchyma and/or cerebellar afferent and efferent pathways as well as cortical, subcortical, and peripheral segments of the central nervous system [3]. Although our understanding of the pathogenesis of different types of CA is continuously growing, so far no medication had been proven effective, except aminopyridines and acetazolamide in episodic ataxia type 2 (EA2) [4, 5]. Because of the general lack of medication, the mainstays of therapy remain physiotherapy, as well as regular occupational and speech therapy [4, 6], but they only have moderate effects on symptom improvement.

In recent years, three case series in different cerebellar ataxia syndromes showed some evidence for the potential efficacy of the modified amino acid acetyl-dl-leucine (AL, usual dosage 3–5 g per day; Tanganil™) with a good overall risk–benefit profile. The first observational study on treatment with AL in 13 patients with degenerative cerebellar ataxia of different etiologies improved ataxic symptoms measured by the Rating of Ataxia Scale (SARA) and Spinocerebellar Ataxia Functional Index (SCAFI) and increased quality of life without any side effects [7]. In patients with Niemann-Pick type C (NPC), the SARA and SCAFI scores also improved; only one patient reported transient dizziness [8]. These findings in NPC were supported by a more recent phase 2 trial [9] and preclinical studies in animal models as well as in gangliosidosis GM2 [10, 11]. The third case series applying AL (500 mg, 4-3-3 tablets per day) in different types of cerebellar ataxia showed improved walking stability in 14 out of 18 patients measured on a GAITRite system [12]. These promising results initiated a recently published multi-center, multinational placebo-controlled crossover trial on the effects of AL in 105 CA patients over six weeks (ALCAT, [13]). The authors concluded, that although the endpoints were not systematically met in this inhomogeneous patient cohort, further symptom-oriented trails evaluating the long-term effects of AL in well-defined subgroups of CA are needed.

To date mechanism and site of action of AL still remain unclear. As was recently shown by physicochemical study acetylation of leucine seems to be a crucial factor so that it can be transported into cells including neurons at a very high rate [14]. An electrophysiological animal study after unilateral peripheral deafferentation in guinea pigs suggested that AL influences the activity of central vestibular nuclei neurons by influencing the channel activity and thereby restoring the membrane potential of depolarized or hyperpolarized vestibular neurons towards normal values [15, 16]. In line with this, an [^18^F]-FDG-µ-PET whole-brain imaging study using a deafferentation rat model showed that AL improves the compensation of postural symptoms in a dose-dependent and specific manner, most likely by activation of the vestibulocerebellum and deactivation of the posterolateral thalamus [17]. Comparable modes and sites of action are assumed for patients with CA, too [18].

It is well-known that the vestibulocerebellum plays an important role in the fine-tuning and timing of sensorimotor integration, serving as a neural integrator system for eye, head, and body movements [19, 20]. In human imaging studies, the critical role of brainstem-cerebellar loops for compensation in central vestibular lesions was supported by an [^18^F]-FDG-PET study in patients with acute unilateral ponto-medullary brainstem infarctions (Wallenberg’s syndrome) affecting the vestibular nucleus by showing signal increases mainly at brainstem-cerebellar level, i.e., in the contralateral medulla and cerebellum [21]. In contrast, in acute unilateral peripheral vestibulopathy and vestibular midbrain infarctions [^18^F]-FDG-PET demonstrated a predominant cortical and subcortical mode of compensatory processes with up- and downregulations primarily within the multisensory vestibular and visual networks [22–24].

This prompted the following questions: First, at which central sites and by which potential mechanisms do central adaptive processes (compensation) take place in patients with CA? Second, in what way is the interaction of brain networks modulated by AL treatment inducing clinical improvement of ataxic symptoms? Our hypothesis was that compensatory processes take place with an upregulation in various cortical sensorimotor networks.

## Subjects and methods

### Patient data and study design

A total of 22 adult patients with cerebellar ataxia (13 females, 9 males, ages 28–77, mean age = 61.9 ± 13.1 years), who received a [^18^F]-FDG-PET imaging of the brain (PET A) without acetyl-dl-leucine (AL) treatment were identified retrospectively from the German Center for Vertigo and Balance Disorders (DSGZ) and the Department of Neurology, University of Munich, Germany (Table 1).Of these 20 received a second PET scan (PET B) with AL (4–5 mg/day) mean 4.7 months later (range 2.5–7.5 months). The mean time interval between the start of AL treatment and the second PET scan was 4.3 months (range 2.3–6.4 months).

**Table 1:** Patient data

Patient	Age/sex [years] [female/male]	Diagnosis	Quality of life [score]	Symptoms duration to PET 1 [years]	Clinical features 1: stance/gait/sitting ataxia 2: ocular motor dysfunction 3: dysarthria 4: limb ataxia	SARA PET 1 [points]	SARA PET 2 [points]	Delta SARA [points]	Responders
1	73 f	MSA-c	0.25	4	1,2,3,4	20	14	− 6	Yes
2	72 f	GAD-antibody encephalitis	0.5	3	1,2,4	16	10.5	− 5.5	Yes
3	28 m	Phenytoin associated CA	0.5	1	1,2,3,4	16	17.5	+ 1.5	No
4	45 f	Ethyl toxic	0.4	5	1,2,3,4	33	22.5	− 10.5	Yes
5	52 f	Ethyl toxic	0.7	3	1,2,3,4	17	14	− 3	Yes
6	75 m	MSA-c	0.3	4	1,2,3,4	12,5	9.5	− 3	Yes
7	42 f	SCA 3	0.6	6	1,2	4,5	2,5	− 2	No
8	61 f	SAOA	0.3	4	1,2,3,4	7	9	+ 2	No
9	69 f	SAOA, small ischaemia	0.2	9	1, 2,3,4	15,5	10	− 5.5	Yes
10	64 f	SAOA	0.3	13	1,2,3,4	12	–	–	–
11	64 m	SAOA	0.4	14	1,2,4	14,5	12	− 2.5	Yes
12	73 m	SAOA	0.8	12	1,2,4	8	8	8.0	No
13	74 f	SAOA with DBN	0.7	3	1,2,4	10,5	–	–	–
14	59 m	SCA 2	0.5	12	1,2,4	16	14.5	− 1.5	No
15	44 m	SCA 1	0.5	6	1,2,3,4	19	15.5	− 3.5	Yes
16	67 f	SAOA mit DBN	0.4	20	1,2,3,4	15,5	13.5	− 2.0	No
17	57 f	SAOA	0.5	7	1,2,3,4	13,5	6	− 7.5	Yes
18	69 m	Puratophin1-gene-mutation	0.6	9	1,2,3,4	12	8.5	− 3.5	Yes
19	74 m	SAOA	–	6	1,2	4,5	1.5	− 3.0	Yes
20	74 m	SAOA	–	5	1,2,4	13	12	− 1.0	No
21	49 f	Multiple sclerosis	0.6	2	1,2,3,4	10	7	− 3.0	Yes
22	77 f	SAOA with DBN	–	5	1,2	3	2	− 1.0	No
*n* = 22	61.9 ± 13.1 y		0.2–0.8	7.0 ± 5.5 y		Mean 13.3	Mean 10.5	Mean 4.5	Responders = 12
	f = 13		Mean 0.48	f = 13					Non-responders = 8
	m = 9								Drop out = 2

*DBN* downbeat nystagmus, *GAD* glutamate acid decarboxylase, *MSAc* multiple system atrophy of cerebellar dysfunction subtype, *SCA* spinocerebellar ataxia, *SAOA* sporadic adult-onset ataxia of unknown aetiology

Patients underwent at least one visit before treatment and at least two follow-up visits to monitor the clinical progress with AL treatment by means of neurological and neurotological examination (including neuro-orthoptic assessment), the health-related five-dimensional Euro-Qol-5D-3L quality of life questionnaire [25], and the standardized Scale for the Rating and Assessment of Ataxia (SARA scale; [26, 27]. The EQ-5D-3L is subdivided into five health state dimensions namely, mobility, self-care, usual activities, pain/discomfort and anxiety/depression, with each dimension assessed in three levels: no problem, some problems, extreme problems. These health states were converted into EQ5D scores using the German time trade-off scoring algorithm [28]. The resulting total EQ5D score ranges from zero to one with higher scores indicating better quality of life. SARA comprises eight items evaluating gait, stance, sitting, speech and limb kinetics, and its score ranges between 0 (no ataxia) and 40 (most severe ataxia level). In addition, patients’ self-assessment of clinical benefit was sampled on medication (improved, no effect, worsened).

The standardized diagnostic procedure revealed the following etiologies of ataxia in the 22 patients: 3 spinocerebellar ataxia (SCA), 1 puratophin1-gene-mutation, 2 multiple system atrophy of cerebellar dysfunction subtype (MSAc), 2 due to chronic alcohol abuse, 1 due to phenytoin consumption, 2 due to inflammatory diseases (1 glutamate acid decarboxylase(GAD)-antibodies, 1 encephalomyelitis), 11 sporadic adult-onset ataxia of unknown etiology (SAOA), one of them with an additional very small cerebellar hemispheric ischemic lesion. All patients showed typical signs of cerebellar dysfunction, e.g., stance/gait/sitting ataxia, dysarthria and/or limb ataxia that lasted between one and twenty years before assignment (mean 7 ± 5.5 years; for details see Table 1). All patients showed typical concomitant ocular motor dysfunction as a sign of central vestibular involvement; five had additional clinical signs of sensory polyneuropathy.

The routinely collected patient data including patient and family history, comorbidities, standardized questionnaires and scores, medication, diagnostic assessments (e.g., clinical, neuro-physiological, laboratory, genetic, imaging), diagnosis, therapy, and outcome were anonymously transferred for descriptive statistical (using SPSS, IBM, Armonk, NY) and subsequent PET analyses (please see below).

In the course of the data evaluation treatment “responders” were defined as showing an improvement of at least 2.5 points in the SARA score accompanied by a subjective benefit. Patients with stable, worsened scores or only minimal improvement (< 2 points) in the SARA score were categorized as “non-responders”.

The individual PET brain images were independently analyzed by two specialists in nuclear medicine (NA, MU) experienced in brain imaging and blinded to the patients. A neuroradiologist reevaluated the MRI scans if an original image was available (12 patients with and 4 patients without cerebellar atrophy). Documentation in the electronic health records or medical discharge letters reported cerebellar atrophy in another 2 patients. Thereby, additional relevant structural MRI lesions that would impair PET image analyses and data interpretation could be excluded in all patients.

### Ethical standards

The study protocol was approved by the Ethics Committee of the University of Munich (21-0166) and was conducted in accordance with the regulations of the Helsinki Declaration.

### PET data analyses

Resting-state PET scanning was performed under identical standardized resting-state conditions with eyes closed in an ECAT EXACT HR + PET or Biograph 64 PET/CT scanner (Siemens Healthcare, Erlangen, Germany) applying 130 ± 20 MBq 2-[^18^F]fluoro-2-deoxy-D-glucose ([^18^F]FDG) intravenously [29, 30]. As it is usually done in clinical [^18^F]-FDG-PET diagnostics, it was possible to exclude pathological cerebral glucose uptake patterns at the cortical level typical of neurodegenerative disorders such as Alzheimer’s disease or other central nervous system disorders.

Group statistical data analysis was performed with Statistical Parametric Mapping software (SPM 8, Wellcome Department of Cognitive Neurology, London, UK, http://www.fil.ion.ucl.ac.uk/spm). For details of PET data preprocessing and statistical analyses (realignment, stereotactical normalization, smoothing, proportional scaling to mean global cerebral activity), please see [21, 23, 24]. To control for normalization effects due to cerebellar hypometabolism, we additionally performed alternative normalization procedures, e.g., to regions of interest sparing the cerebellum, in the thalamus and frontal white matter, which gave very similar results. We decided to report the results of the standardized voxel-wise procedure to overcome a priori knowledge bias inherent in the semiquantitative assessment using the region of interest approach [31] and to ensure optimal data comparability to those of earlier patient [^18^F]-FDG-PET studies. Finally, the following t-statistical parametric maps were calculated: (1) statistical comparison between the whole patient group and sex- and age-matched healthy control group by a two-sample t-test (*n* = 20; mean age = 61.0 ± 0.9 years; no history or signs of former vestibular dysfunction), (2) statistical comparison between the responders resp. non-responders and the healthy control group separately, (3) direct subtraction analyses of the two patient PET scans by a paired t test (PET A vs. PET B; off-treatment vs on-treatment) for the whole patient group, as well as for the responders and non-responders separately, (4) direct subtraction analyses of treatment responders vs. non-responders, (5) statistical correlation analyses between [^18^F]-FDG uptake and different clinical and behavioral parameters e.g., disease duration, ataxia (SARA) and quality of life scores in the questionnaires. The comparison with a healthy control group was necessary since no PET scan prior to disease onset was available for the patients. For the correlation analyses, the individual clinical parameters belonging to each PET image were entered as covariates into the design matrix. Furthermore, all contrast data were corrected for age by entering the individual ages as covariates into the analyses.

Regions were identified using an atlas machine referring to the Jülich Histological Atlas, the Harvard–Oxford Cortical and Subcortical Structural Atlases, and the MNI Structural Atlas (http://ftp.nmr.mgh.harvard.edu). The single components of cerebellar clusters were elaborated with caution and described only roughly to their position because of the limited spatial resolution of PET.

Clusters were considered significant for *p* ≤ 0.001 uncorrected and if larger than ten voxels. For illustrative purposes, partially clusters above a threshold of *p* ≤ 0.005 are added to the text according to the theory-driven a priori hypothesis for visual, vestibular and somatosensory areas [21–24, 32].

## Results

### Patient clinical data

Data from 22 CA patients could be enrolled for off-treatment PET analyses, 20 additionally for on-treatment PET analyses. Overall, the SARA scores ranged between 3 and 33 points before treatment (mean 13.3 points), and between 2 and 22.5 points (mean 10.5 points) on treatment (Table 1). The 12 responders improved with treatment by a mean of 4.5 points in the SARA score. Eight patients were identified as not significantly improving with treatment measured by the SARA (≤ 2 points). They all judged themselves as unimproved on medication and thus were classified as non-responders. The SARA score before treatment was mean 15.6 ± 7.1 points in the responders and 10.4 ± 9.2 points in the non-responders. Thus, before treatment, the later on identified responders tended to be more affected by cerebellar ataxia (*p* = 0.055).

### PET calculations for the whole group of CA patients

#### Patients PET A (before treatment) vs. healthy controls

**Fig. 1:**
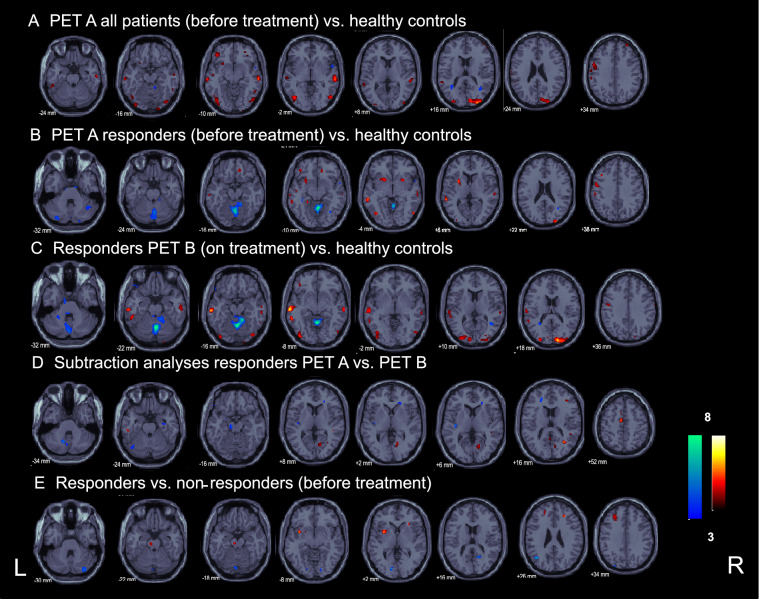
Statistical group analyses of FDG-PET in CA patients. Results of the different group analyses of the [^18^F]-FDG-PET scans in CA: **A** for all patients, before treatment, **B,** respectively, the AL treatment “responders” only before as well as **C** on treatment compared to a healthy control group, **D** direct categorical comparisons of the two patient [^18^F]-FDG-PET scans (before vs. on treatment and vice versa) for the AL treatment “responders” subgroup, and **E** results of the direct subtraction analyses of “treatment responders” vs. “non-responders” before treatment with AL (*p* ≤ 0.001)

The contrast showed widespread bilateral positive signal changes in parts of the occipital pole and lateral occipital cortex according to primary and secondary visual cortex areas (V1–V5, including motion-sensitive areas) as well as in the posterior division of the middle/superior temporal gyri (BA 20/22) that partly merge into the inferior temporal gyri or show separated clusters there. On the left side, there were further positive clusters in the central sulcus region, precentrally (primary motor) as well as postcentrally (somatosensory) and in the left anterior insula cortex. Smaller clusters were seen in the fronto-orbital cortex/pole (Fig. 1, Supplementary Table 1). By lowering the threshold to *p* < 0.005, signal changes in the areas described above became larger in size and more symmetrical, but no functionally relevant new areas showed up.

The inverse contrast of healthy controls compared to patients before treatment gave small clusters in the right cerebellum, the right superior frontal gyrus, as well as in the optic radiation and the parahippocampal gyrus bilaterally (Fig. 1, Supplementary Table 1). With lowered threshold the negative signal changes in the patients compared to healthy controls showed up in several parts of the cerebellum (at midline and in both hemispheres), but no new cortical sites occurred.

#### Patients PET B (on treatment) vs. healthy controls

This contrast showed similar positive signal changes in the patients on treatment compared to healthy controls bilaterally in the occipital pole and lateral occipital cortex according to primary and secondary visual cortex areas V1/V2–V5 (including motion-sensitive area) as well as in the posterior division of the middle temporal gyrus, partly merging into and partly separated to clusters in the inferior / superior temporal gyri (Supplementary Fig. 1, Supplementary Table 2). On both sides, further clusters were located in the postcentral gyrus (somatosensory) and on the left a small cluster in the primary motor area. Smaller signal changes were seen in the left frontal cortex and the left putamen.

The inverse contrast of healthy controls compared to patients on treatment showed at the standard threshold a large midline cerebellar cluster with its maximum in the cerebellar vermis. Further smaller clusters were located in the right thalamus, the right parahippocampal gyrus, the right temporo-occipital part of the middle temporal gyrus and the left optic radiation/callosal body (Supplementary Fig. 1, Supplementary Table 2).

By lowering the threshold, all clusters became larger in size and more symmetrical, but no functionally relevant additional areas showed up. Negative signal changes in the patients compared to healthy controls were now widespread in both cerebellar hemispheres and midline structures.

In summary, the rCGM pattern in the patients with and without treatment compared to healthy controls appeared to be very similar, mainly showing common signal increases in the bilateral visual (V1/2, V5), temporo-parietal vestibular and somatosensory/premotor cortex, while cerebellar signal decreases showed up only with treatment or with a lowered threshold. No newly activated areas showed up on treatment.

Direct subtraction analysis of patients PET B (on treatment) vs. PET A (before treatment):

The direct contrast between PET B and PET A showed at regular threshold only one very small cluster in the left supramarginal gyrus (10 voxels), and the inverse contrast (PET A vs. PET B) in the left cerebellar hemisphere (80 voxels), the right temporal fusiform/parahippocampal gyrus (49 voxels), and superior parietal lobule/postcentral gyrus (14 voxels) (Supplementary Fig. 1, Supplementary Table 3). Only by lowering the significance level, bilateral clusters showed up in the superior frontal gyrus/juxtapositional lobule cortex (premotor cortex BA 6 left > right), the superior parietal lobules/intraparietal sulcus (BA 7), and supramarginal/middle temporal gyrus (partly somatosensory) (PET B vs. PET A). Further, unilateral cortical clusters were mainly located in the right visual cortex (V1, BA17)/optic radiation and the posterior callosal body. At the cerebellar level there was still only one small cluster most probably located in right lobule VI / nodulus (19 voxels). The inverse contrast PET A vs. PET B gave small clusters in parts of the cerebellar hemispheres/cruses and the posterior insular cortex bilaterally, and unilaterally in the right middle/superior temporal gyrus, the right temporal fusiform/parahippocampal gyrus, the right central sulcus region post-/precentrally, partly premotor cortex, and the right occipital pole and lateral occipital cortex.

In summary, for the whole patient group the direct subtraction analyses between the two PET scans before and on treatment with AL revealed only minor differences at the standard significance level. Only with lowered threshold small bilateral clusters showed up at the cortical level (primarily in the secondary somatosensory, visual cortex, precuneus, parts of the temporo-parietal vestibular network, and premotor cortex) due to increased rCGM with treatment. At the cerebellar level there were mainly signal decreases with treatment.

### PET calculations for treatment responders

#### Responders PET A (before treatment) vs. healthy controls

This contrast gave clusters primarily in parts of the occipital pole and lateral occipital cortex bilaterally according to primary and secondary (motion-sensitive) visual cortex areas V1, V2 and V5. Further clusters were located in the middle/superior temporal gyri (partly BA 21 vestibular), and putamen/pallidum bilaterally (left > right). In the left hemisphere, there were further activation clusters in the central sulcus region pre-/postcentrally (primary motor/premotor/somatosensory), and the parahippocampal gyrus. Smaller signal differences were seen in the frontal cortex bilaterally (Fig. 1, Supplementary Table 4).

The inverse contrast of healthy controls compared to responders before treatment showed significant signal differences in midline structures as well as bilateral hemispheric cerebellar regions partly including the cerebellar cruses. Small clusters projected most probably onto the left optic radiation/callosal body, the right inferior parietal lobule and the left hippocampus.

By lowering the threshold, the positive clusters in the patients’ PET A compared to healthy controls became larger in size and more symmetrical, but no functionally relevant new areas were found. The negative signal changes in the cerebellum now merged into the medullary brainstem. Supratentorial clusters were localized in the posterior cingulate gyrus, the posterior callosal body/optical radiation bilaterally, and a small cluster in the right middle temporal gyrus/temporal pole.

#### Responders PET B (on treatment) vs. healthy controls

On treatment the patients also showed bilateral widespread activation in the occipital pole and lateral occipital cortex according to primary and secondary visual cortex areas V1, V2, V4 and V5 bilaterally (including motion-sensitive areas) that merged upward into the parietal lobule/precuneus. Further bilateral clusters were found in the middle/superior temporal (mostly posterior division, partly BA 21 vestibular), and postcentral gyri (somatosensory cortex) partly reaching the precentral gyri (primary motor) (Fig. 1, Supplementary Table 5). By lowering the significance level, all these areas became larger in size and more symmetrical. New small clusters showed up in the right posterior/retroinsular region (multisensory vestibular cortex) as well as in the putamen/ caudate nucleus bilaterally (Supplementary Fig. 2).

The inverse contrast showed distinct negative signal differences compared to healthy controls predominantly in the patients’ cerebellar vermis and both cerebellar hemispheres, the midline vermal cluster of which also survived correction for multiple comparisons (Fig. 1, Supplementary Table 5). Supratentorial clusters were rare even with lowered threshold despite one midline cluster in the right juxtapositional lobule (supplementary motor cortex), a small cluster in the right middle temporal gyrus, as well as in the intraparietal sulcus/callosal body. With lowered threshold these negative signal changes, especially in the cerebellum (and newly brainstem) and juxtapositional lobule (supplementary motor cortex) became larger in size. No new negative clusters showed up.

#### Direct subtraction analysis of responders PET B (on treatment) vs. PET A (before treatment)

The direct contrast between the PET B and A showed clusters in the right post/precentral gyrus (somatosensory/premotor, 17 voxels), the left inferior temporal gyrus (12 voxels), and the left juxtapositional lobule cortex (formally supplementary motor cortex, 43 voxels) that all became bilateral and larger in extent with a lowered threshold (Fig. 1, Supplementary Table 6). Additionally, in this setting several smaller clusters in occipital predominantly secondary (lateral occipital cortex/precuneus) rather than primary visual cortex areas (lingual gyrus, cuneus) were seen.

The inverse direct contrast PET A vs. PET B showed small signal differences (due to lowered rCGM with treatment) in the midline cerebellum (15 voxels), premotor area (13 voxels), the anterior callosal body (11 voxels) and the left posterior insular cortex (vestibular, 11 voxels) at *p* < 0.001. The latter all became larger in size with lowered threshold and then comprised at infratentorial level potentially parts of the vermis, left VIIIa, VIIb, and Crus I/II. With the lowered threshold additional small clusters in the midbrain/pontine brainstem, the (para-)hippocampal gyrus/amygdala bilaterally, and the anterior callosal body occurred.

In summary, responders showed positive (primary and secondary visual cortex, parts of the temporo-parietal vestibular network, somatosensory, and premotor cortex) as well as negative clusters (chiefly within the midline cerebellum), which were located at similar sites as in the whole patient group. The pattern of upregulation and downregulation appeared considerably pronounced in the responders with treatment. This was especially true for the negative cerebellar midline vermal cluster that even survived correction for multiple comparisons with treatment (PET B).

### PET calculations for treatment non-responders and comparisons of responders versus non-responders

Compared to healthy controls, the non-responders showed a similar, but less pronounced pattern of upregulation of brain metabolism in cortical areas belonging to the visual, somatosensory, and vestibulo- temporo-parietal cortical networks, and downregulation in the cerebellar midline (vermal) and hemispherical areas than the responders and the whole patient group did. Direct subtraction analyses between PET B and PET A in non-responders revealed no significant differences in rCGM, even at lowered threshold (in line with the missing clinical benefit).

Calculation of treatment responders vs. non-responders before treatment (PET A) gave clusters in the left mid- and right anterior insular cortex, in the pontine brainstem, and the middle fontal gyri bilaterally, whereas the inverse contrast of non-responders vs. responders gave clusters in both visual cortices (V1–V3), partly merging into the precuneus/parietal lobules and lateral occipital parts (Fig. 1). With treatment (PET B) the contrast between responders and non-responders showed no significantly increased rCGM, and only two decreased rCGM clusters in the juxtapositional lobule cortex (supplementary motor cortex), and most probably in the visual cortex (lingual cortex/precuneus). Due to the differences in sample sizes (9 non-responders vs. 13 responders), we do not report and discuss these anatomical sites in more detail.

### Correlation analyses with clinical parameters

**Fig. 2:**
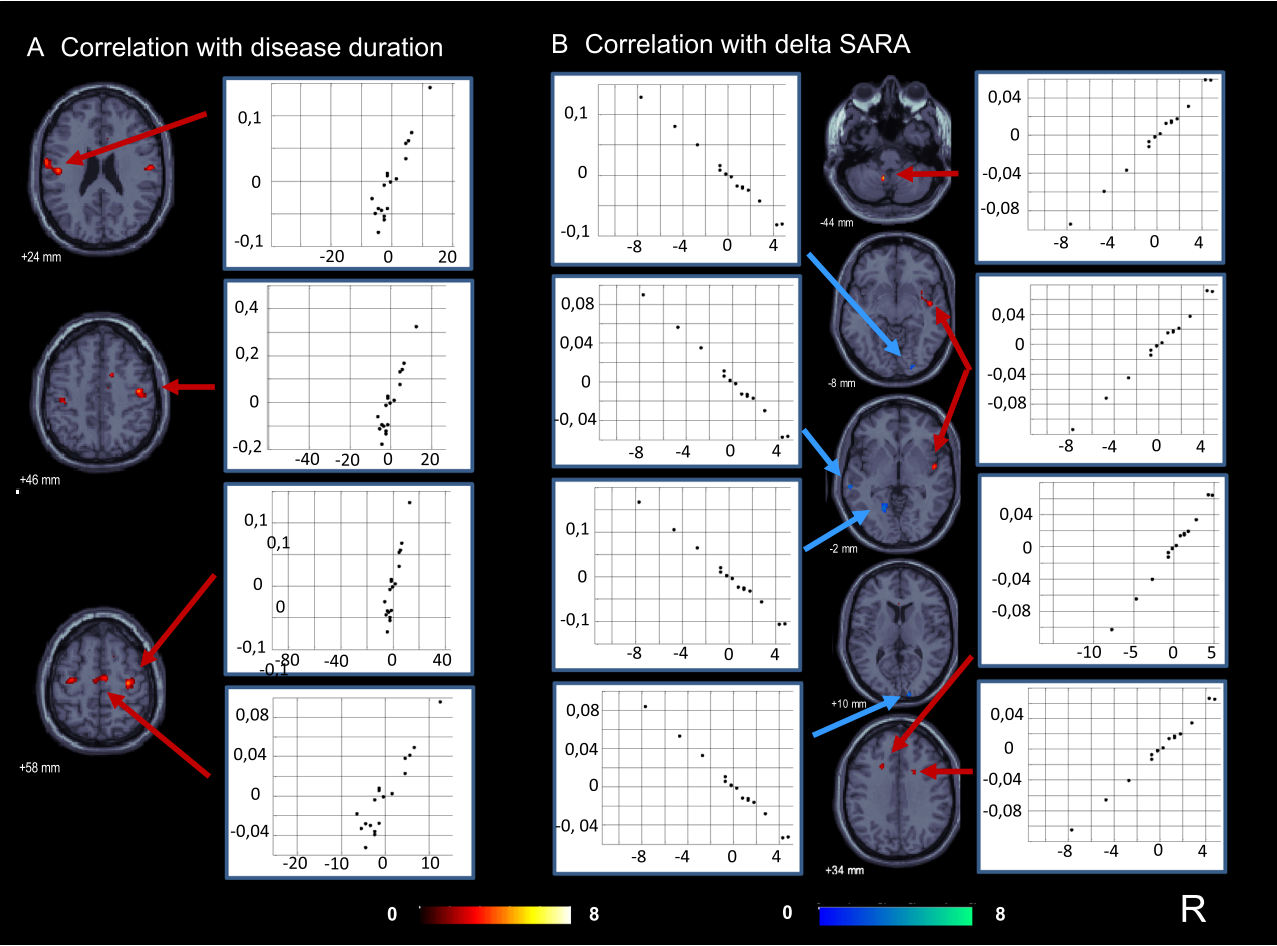
Correlation analyses with disease duration and improvement of ataxia with AL treatment. Before treatment (PET A) the duration of cerebellar symptoms was found to be positively correlated with the glucose metabolism (the longer the symptoms lasted, the higher the metabolism) in the central sulcus region bilaterally, including primary and secondary somatosensory as well as primary/premotor cortex areas (juxtapositional lobule cortex; formally supplementary motor cortex). **B** The improvement of ataxia with AL treatment (decreasing SARA score) was correlated with a decrease in glucose metabolism between PET A and B (indicated in blue) in bilateral parts of the visual cortex and the superior/middle temporal gyrus (secondary vestibular network area), as well as with an increase in glucose metabolism (indicated in red) in the midline left cerebellum and the right posterior insula region (multisensory vestibular)

Without treatment (PET A) the quality of life in CA patients showed a positive correlation with the rCGM in the widespread midline as well as bilateral hemispherical cerebellar regions (the higher the QoL the higher the metabolism) (Supplementary Fig. 2), which was not the case for the duration of cerebellar symptoms. The latter instead showed a symmetrical widespread positive correlation with rCGM (the longer the symptoms last, the higher the metabolism) in the central sulcus region bilaterally, including primary and secondary somatosensory (BA 2/3, OP 4) as well the primary/premotor cortex areas (BA 4/6, juxtapositional lobule cortex; formally supplementary motor cortex) (Fig. 2).

The improvement of ataxia with treatment (decreasing SARA) correlated with the decrease in rCGM between PET A and B in parts of the visual cortex bilaterally and the superior/middle temporal gyrus (secondary vestibular network area), as well as with the increase in rCGM in the midline left cerebellum (most probably tonsil), the right posterior/middle insula region (vestibular), the upper right premotor cortex/cortico-spinal tract, and anterior parts of the callosal body (Fig. 2).

## Discussion

Compared to healthy controls, the cerebral glucose metabolism in the whole CA patient group was found to be lowered at the cerebellar (midline vermal and hemispherical) level, and increased at the cortical level in several sensory areas such as somatosensory (postcentrally), visual in the temporo-parietal-occipital cortex (e.g., primary and secondary visual areas including motion-sensitive area V5 and the precuneus), as well as secondary vestibular (e.g., middle/superior temporal gyrus, anterior insula). Additionally, rCGM was increased in the juxtapositional lobule cortex and precentral gyrus representing supplementary and primary motor areas. Subgroup analyses for responders and non-responders confirmed this robust pattern of bilateral signal increases in multisensory-motor cortex areas, and signal decreases at the cerebellar level that was especially pronounced with AL treatment in the responder subgroup. However, direct subtraction analyses between both [^18^F]-FDG-PET scans before and with AL treatment gave significant differences only for the treatment responders.

The vestibulocerebellum plays an important role in the fine-tuning and timing of sensorimotor integration not only of semicircular canal and otolith information from the vestibular periphery, but also from other sensory systems (somatosensory, optokinetic, visual, and neck proprioceptive). Together with the vestibular nuclei complexes in the brainstem—which phylogenetically belong to the cerebellum—it serves as a neural integrator system for eye, head, and body movements [19, 20]. In the current CA patient cohort, the involvement of the vestibulocerebellum was reflected by dysfunction of stance and gait and the presence of cerebellar ocular motor dysfunction in all patients, e.g. downbeat nystagmus, gaze-holding deficit or saccadic smooth pursuit [33–36].

This upregulation of multisensory cortical network areas in CA is not surprising since a predominant cortical path of compensatory processes by up- and downregulations in visual-vestibular networks was found in earlier human [^18^F]-FDG-PET studies in acute unilateral peripheral vestibulopathy as well as acute vestibular midbrain infarctions [22–24]. On the other hand, after vestibular nucleus lesions, compensation occurred preferably via contralateral brainstem-cerebellar loops and largely spared cortical areas [21]. This interesting finding was explained by readjusting integration processes via the intact contralateral brainstem and vestibulocerebellum. However, the anatomical and functional suppositions in CA are different to unilateral peripheral vestibulopathy or vestibular nucleus lesions: All structures from the peripheral vestibular endorgans via vestibular brainstem centres and bilaterally ascending vestibular pathways [33, 37] were not primarily affected by the cerebellar disease, and thereby bilateral signal transmission to the cortex should not be impaired, but tuning and timing of sensorimotor integration was disturbed.

In agreement with the above-cited resting-state [^18^F]-FDG-PET studies in different central- and peripheral vestibular lesions, even in the current study the patients were lying still in the scanner with eyes closed and without any movement or postural task. Thus, rCGM changes reflect tonic changes in brain metabolism and sustained central plasticity processes. They can be interpreted as tonic shifts towards multisensory and (pre-)motor brain network areas to recruit optimal sensory information for better balance, body movement and ocular motor control. Interestingly, not only primary but also secondary visual areas of the temporo-parietal-occipital network (precuneus/superior parietal lobule, motion-sensitive visual middle temporal area MT) showed rCGM upregulation. MT/V5 is a region of the extrastriate visual cortex that contains a high concentration of direction-selective neurons and is thought to play a major role in the perception of visual motion, the integration of local motion signals into global percepts as well as in the guidance of certain eye movements [38]. The region of the precuneus/superior parietal lobule plays a key role in visual cognition, spatial attention, and imagery as well as working memory, and especially the precuneus shows increased functional connectivity with a distributed network of parietal, occipital, and temporal neocortical regions (including V2 and V3) [39], thereby representing a potential hub region that mediates the integration of distributed visuospatial information into a more holistic representation [40]. In line with these anatomical and functional interconnections, the precuneus was also identified as part of the ensemble of areas belonging to the multisensory vestibular network [41], to which the superior temporal gyrus that was found to be bilaterally upregulated in CA also belongs. Summing up, reinforced usage and integration of somatosensory, primary and secondary visual and vestibular cortical network areas appears useful in CA to compensate for unbalanced vestibular and ocular motor signals due to chronic cerebellar dysfunction. In line with our findings, a retrospective [^18^F]-FDG-PET study in 17 genetically-confirmed SCA 3 patients also reported relatively increased metabolism in somatosensory and vestibular insula areas [42]. Due to concomitant hypometabolism not only in the cerebellum but also in the posterior parietal cortex including the precuneus these increases were interpreted as changes in coherent cerebellar-parietal functioning and a consequence of impaired forward processing in higher-order motor control. In contrast to our patient cohort, SCA 3 patients typically develop additional pyramidal and extra-pyramidal signs, neuropathy and cognitive problems, which were not systematically present here.

rCGM increases in the precentral gyrus and premotor cortex, partly the formally supplementary motor cortex, might reflect the increased use of efferent motor pathways especially with AL treatment projecting to the spinal cord to better posturally stabilize and control the body, bimanual actions and movements that are internally generated.

**Fig. 3:**
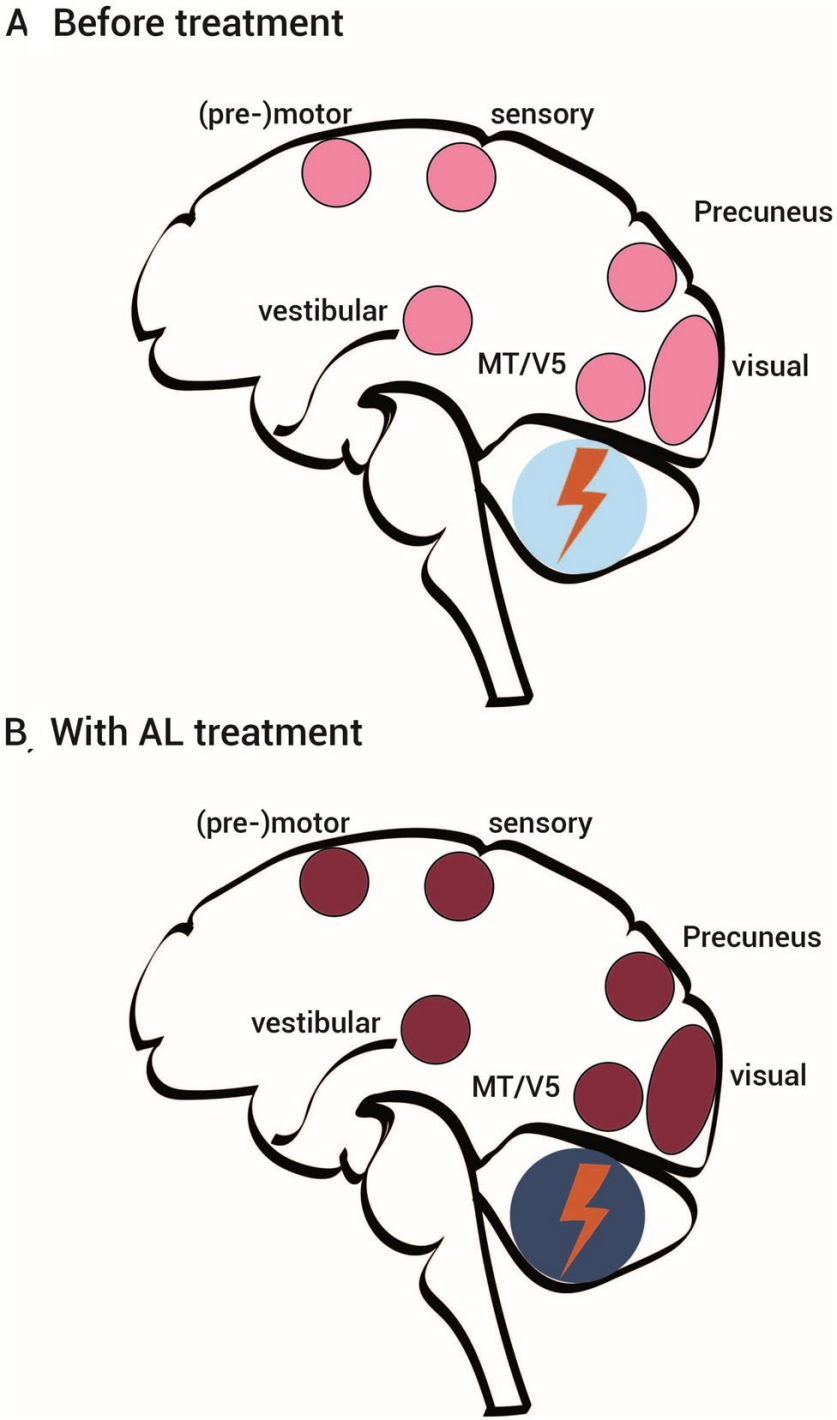
Schematic drawing. Schematic drawings of rGCM-increases (red) and rCGM-decreases (blue) in patients with chronic cerebellar ataxia, **A** before treatment and **B** with treatment with acetyl-d-leucine (AL) according to the current study results of resting-state [^18^F]-FDG-PET imaging with eyes closed. The visual (primary and secondary visual cortex including MT/V5, precuneus) and vestibular network areas (insular cortex, superior temporal gyrus, superior parietal lobule) were found to be upregulated, as were somatosensory as well as primary and secondary motor cortex areas (juxtapositional lobule cortex and precentral gyrus) bilaterally, while glucose metabolism was decreased at cerebellar (midline vermal and hemispherical) level. With AL the divergence of cortical sensorimotor upregulation and cerebellar downregulation was enhanced (indicated by darkened red and blue colors)

In general, decreased cerebellar [^18^F]-FDG uptake is a typical finding in inherited and sporadic chronic CA at least in the advanced phases with structural cerebellar damage [31, 43]. However, the correlation between glucose metabolism deficit and severity of clinical cerebellar deficit often varies. It is important to note that in our study cerebellar hypometabolism was evident in 14 out of the 22 CA patients (no patient showed increased tracer uptake), but without systematic co-occurrence with cerebellar atrophy in structural MRI. Consequently, group-level statistics revealed cerebellar hypometabolism only with lowered significance level. However, in the responder subgroup predominant cerebellar midline vermal clusters were evident already at standard thresholds and even survived correction for multiple comparisons with treatment. Thus, the measurable effect of AL on the cortical activation-deactivation pattern was a strengthened divergence between cortical sensorimotor rCGM increases described above and deepened cerebellar(-brainstem) decreases, that was pronounced in responders (Fig. 3).

One mechanism of action of AL is that it normalizes membrane potential and neuronal excitability as it was found in vestibular nuclei neurons [15, 16, 18]. The only available brain imaging study on central effects of AL used a peripheral deafferentation rat model and [^18^F]-FDG-µ-PET [17]. The authors reported an improved compensation of postural symptoms in a dose-dependent and specific manner with AL (with the L-form being the active isomer), mostly likely by activation of the vestibulocerebellum and deactivation of the posterolateral thalamus. This is in contrast to the findings in our current study, which showed no increase of rCGM in the cerebellum not even with lowered threshold, but a deepened rCGM decrease and no thalamic signal changes. However, unlike our CA patients with a chronic disorder, in acute peripheral deafferentation the integrating brainstem-cerebellar function is not impaired but used for compensation [22, 23]. Consequently, influences on membrane potential and excitability of vestibular nuclei neurons in the brainstem by AL treatment must inevitably lead to different brain activation patterns.

Interestingly, the responders tended to show higher mean SARA scores before treatment than the non-responders (15.6 vs. 10.4 points). Thus, one could speculate, that if a treatment effect is noticeable for the patients it is only measurable in advanced stages of CA. However, one should be cautious with such general statements, since the number of patients in our study is rather limited and standard deviations of SARA scores were relatively high, even in the responders subgroup showing a pronounced cerebellar hypometabolism.

The method of PET imaging of course is not suitable to carve out the exact site of action of a medical treatment such as AL. However, the findings of a positive correlation between QoL before treatment with the rCGM in the midline as well as bilateral hemispherical cerebellar regions (the higher the QoL the higher the metabolisms), and the positive correlation between the improvement of ataxia with AL treatment (decreasing SARA) and increasing rCGM between the two PET scans in the midline cerebellum points towards a mechanism enhancing the cerebellum’s inhibitory control function [44]. Notably, cerebellar hypometabolism does not inevitably stand for a generally reduced cerebellar function but can also reflect inhibitory mechanisms. Thus, an amplification of inhibitory mechanisms by AL might explain the deepened cerebellar hypometabolism with treatment, especially in responders.

Correlation analyses further underlined the pivotal role of multisensory cortical compensation mechanisms by shifts towards sensorimotor pathways/loops in CA in two findings: First, ataxia improvement with treatment (improved SARA score) correlated with the increase in glucose metabolism in parts of the vestibular cortex (posterior insula) and premotor cortex, and second, the duration of symptoms showed positive correlation with the rCGM in the superior temporal gyrus (secondary vestibular), precentral/postcentral and supplementary motor areas (motor circuit). This is an interesting aspect pointing towards potential physical therapy programs aiming for intensified multisensorimotor integration (visual and somatosensory) and addressing the intentional use of sensory input for optimized motor control in CA patients.

The main shortcoming of our study is its retrospective approach including a heterogeneous CA patient collective. We included only CA patients who were well-diagnosed, monitored and documented in house. Even so, the original structural MRI data were not available anymore in all patients for direct overlay with PET and perfectly correlate cerebellar atrophy and hypometabolism. However, no systematical co-occurrence of metabolism and atrophy was found, and a relevant change of cerebellar metabolism induced by AL (as found here) could not be expected in significant cerebellar atrophy. Moreover, the whole patient group did not show significant hypometabolism before treatment compared to healthy controls that could be easily attributed to atrophy. The number of patients for subgroup analyses were small, but results could be described at a similar significance level and group sizes, making them comparable to those in earlier [^18^F]-FDG-PET studies on other vestibular disorders [21–24]. Furthermore, we avoided detailed discussion on single cluster localization and focused on a more global concept of cerebral interaction of areas and mechanisms of compensation in CA.

In summary, in the current PET imaging study we explored compensation processes of the human brain in chronic cerebellar dysfunction and the sites of action of AL. A chronic disorder of cerebellar integration—with an intimate interplay with brainstem circuits—leads to compensatory activation of several sensorimotor systems at the cortical level, while brain metabolism in the cerebellum itself is downregulated (probably due to disturbing cerebellar input). In some patients, treatment with AL was able to improve compensatory mechanisms, i.e., further increase of activation at the cortical level, and further decrease in the cerebellum (Fig. 3).

## Data availability statement

Data are available on request from the corresponding author.

## Supplementary Information

Below is the link to the electronic supplementary material.

Supplementary Fig. 1: Results of (A) the statistical group analyses of the second [18F]-FDG-PET scans on AL treatment of all CA patients compared to a healthy control group, as well as of the subtraction analysis of both [18F]-FDG-PET scans (before vs. on treatment and vice versa) thresholded at p≤0.001. (PPTX 1134 KB)

Supplementary Figure 2: Before treatment the quality of life in CA patients showed a positive correlation with the glucose metabolism in midline as well as bilateral hemispherical cerebellar regions (the higher the QoL the higher the metabolism) (PPTX 1298 KB)

Supplementary Table 1: Whole patient group: Areas showing significant differences in glucose metabolism in the statistical subtraction analyses thresholded at p≤0.001 uncorrected for CA patients’ PET A (before treatment) vs. healthy controls and vice versa. Supplementary Table 2: Whole patient group: Areas showing significant differences in glucose metabolism in the statistical subtraction analyses thresholded at p≤0.001 uncorrected for CA patients’ PET B (on treatment with AL) vs. healthy controls and vice versa. Supplementary Table 3: Areas showing significant differences in glucose metabolism in the direct subtraction analyses of PET A (before treatment) and B (on AL treatment) (A vs. B and vice versa). Supplementary Table 4: Subgroup analyses only in AL treatment “responders”: Areas showing significant differences in glucose metabolism in the statistical subtraction analyses thresholded at p≤0.001 uncorrected CA patients’ PET A (before treatment) vs. healthy controls and vice versa. Supplementary Table 5: Subgroup analyses only in AL treatment responders: Areas showing significant differences in glucose metabolism in the statistical subtraction analyses thresholded at p≤0.001 uncorrected for CA patients’ PET B (on treatment with AL) vs. healthy controls and vice versa. Supplementary Table 6: Areas showing significant differences in glucose metabolism in the direct subtraction analyses of “treatment responders” vs. “non-responders” (A vs. B and vice versa). (PDF 891 KB)
