# Supplementary material for: Acetyl-dl-leucine in cerebellar ataxia ([18F]-FDG-PET study): how does a cerebellar disorder influence cortical sensorimotor networks?
Source: J Neurol. 2022 Jul 25;270(1):44–56. doi: 10.1007/s00415-022-11252-2 (PMC9813104; doi:10.1007/s00415-022-11252-2)
Supplement: Supplementary file 1 — Supplementary Fig. 1: Results of (A) the statistical group analyses of the second [18F]-FDG-PET scans on AL treatment of all CA patients compared to a healthy control group, as well as of the subtraction analysis of both [18F]-FDG-PET scans (before vs. on treatment and vice versa) thresholded at p≤0.001. (PPTX 1134 KB) [file 415_2022_11252_MOESM1_ESM.pptx]

## Slide 1
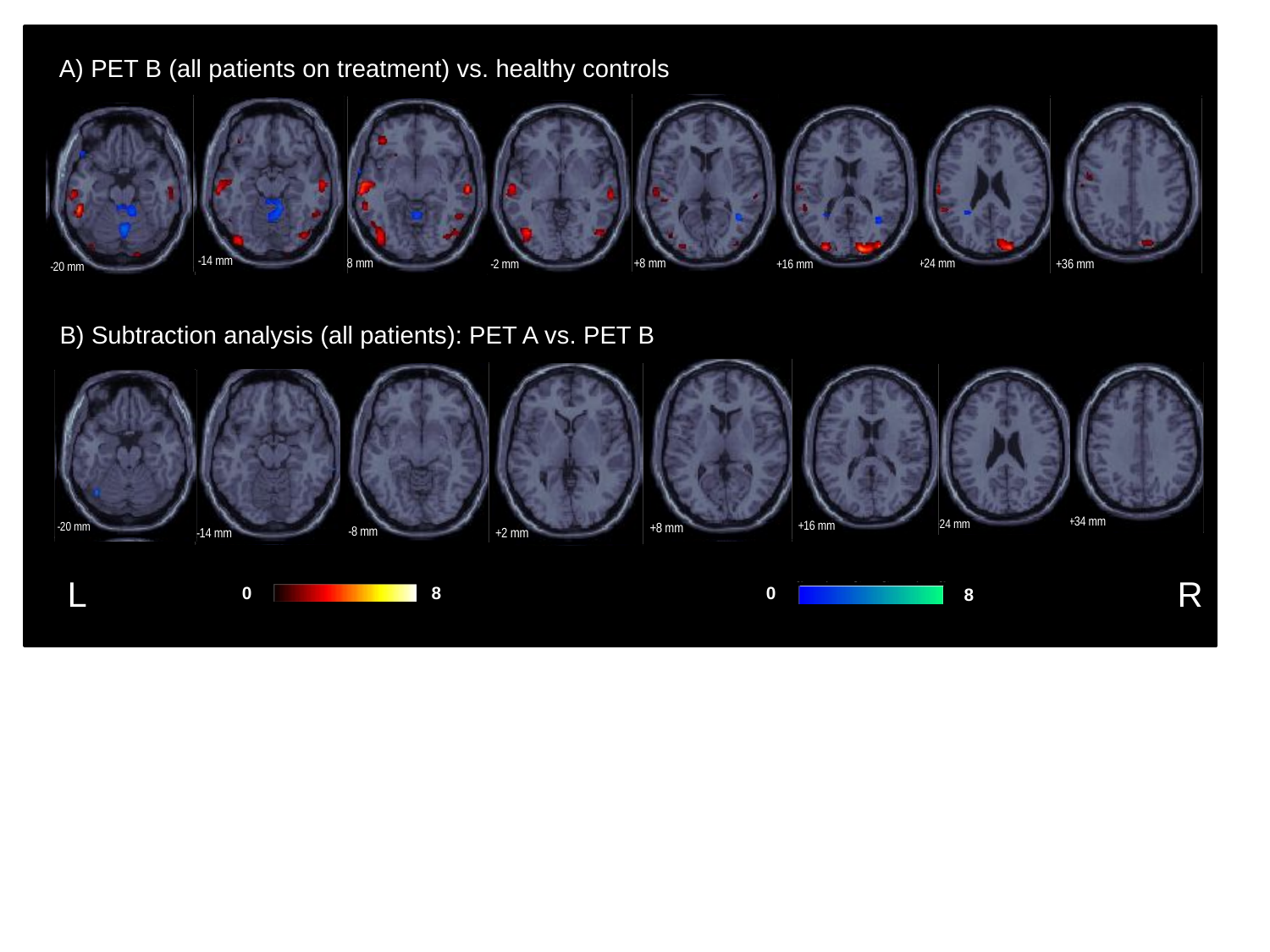

A) PET B (all patients on treatment) vs. healthy controls
B) Subtraction analysis (all patients): PET A vs. PET B
L
R
 0 8
0
8
