# Supplementary figures and images for: Acetyl-dl-leucine in cerebellar ataxia ([18F]-FDG-PET study): how does a cerebellar disorder influence cortical sensorimotor networks?
Source: J Neurol. 2022 Jul 25;270(1):44–56. doi: 10.1007/s00415-022-11252-2 (PMC9813104; doi:10.1007/s00415-022-11252-2)

## Slide 1
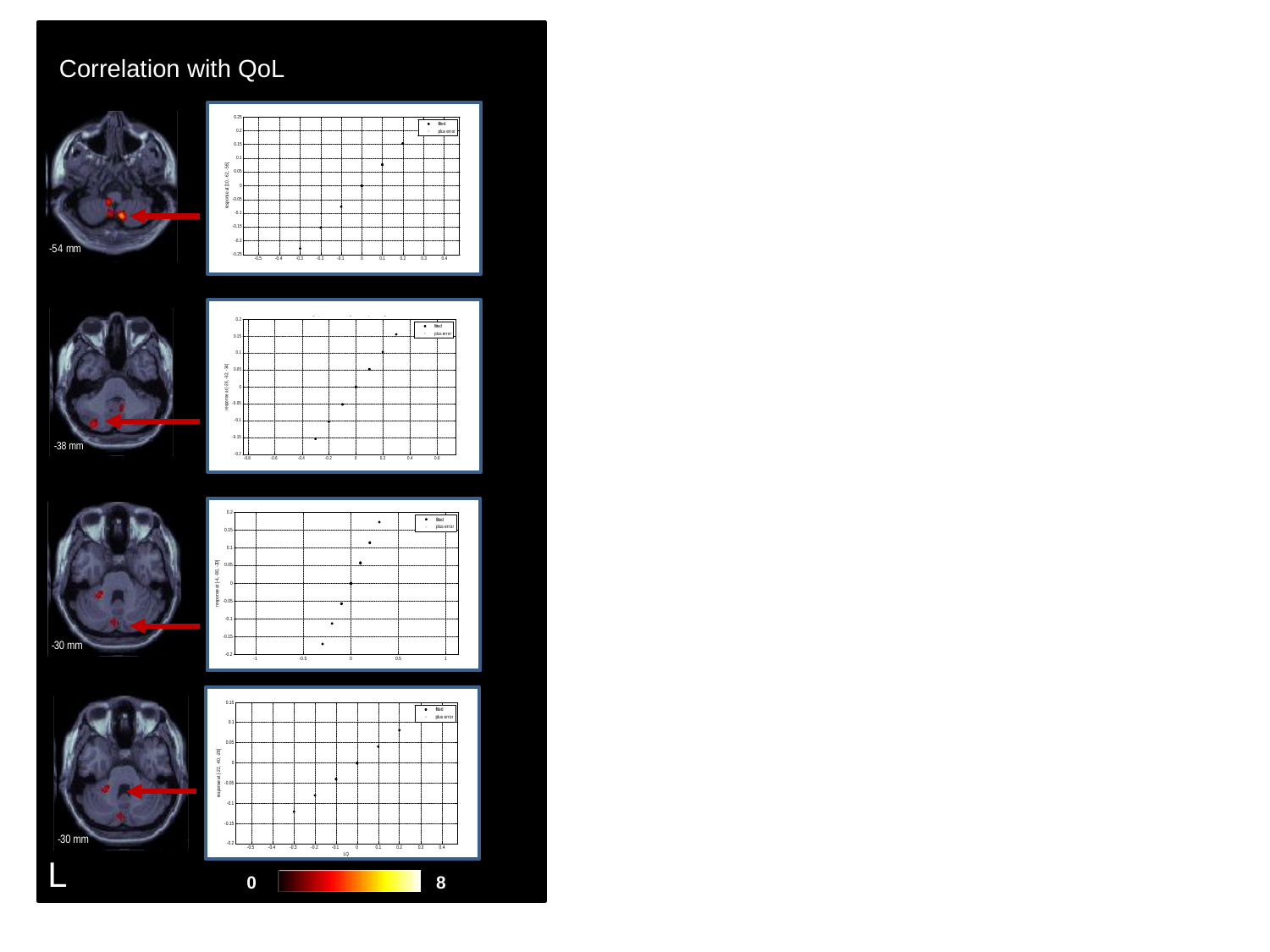

Correlation with QoL
L
 0 8

Supplement: Supplementary file 2 — Supplementary Figure 2: Before treatment the quality of life in CA patients showed a positive correlation with the glucose metabolism in midline as well as bilateral hemispherical cerebellar regions (the higher the QoL the higher the metabolism) (PPTX 1298 KB) [file 415_2022_11252_MOESM2_ESM.pptx]
