# Supplementary material for: Acetyl-dl-leucine in cerebellar ataxia ([18F]-FDG-PET study): how does a cerebellar disorder influence cortical sensorimotor networks?
Source: J Neurol. 2022 Jul 25;270(1):44–56. doi: 10.1007/s00415-022-11252-2 (PMC9813104; doi:10.1007/s00415-022-11252-2)
Supplement: Supplementary file 3 — Supplementary Table 1: Whole patient group: Areas showing significant differences in glucose metabolism in the statistical subtraction analyses thresholded at p≤0.001 uncorrected for CA patients’ PET A (before treatment) vs. healthy controls and vice versa. Supplementary Table 2: Whole patient group: Areas showing significant differences in glucose metabolism in the statistical subtraction analyses thresholded at p≤0.001 uncorrected for CA patients’ PET B (on treatment with AL) vs. healthy controls and vice versa. Supplementary Table 3: Areas showing significant differences in glucose metabolism in the direct subtraction analyses of PET A (before treatment) and B (on AL treatment) (A vs. B and vice versa). Supplementary Table 4: Subgroup analyses only in AL treatment “responders”: Areas showing significant differences in glucose metabolism in the statistical subtraction analyses thresholded at p≤0.001 uncorrected CA patients’ PET A (before treatment) vs. healthy controls and vice versa. Supplementary Table 5: Subgroup analyses only in AL treatment responders: Areas showing significant differences in glucose metabolism in the statistical subtraction analyses thresholded at p≤0.001 uncorrected for CA patients’ PET B (on treatment with AL) vs. healthy controls and vice versa. Supplementary Table 6: Areas showing significant differences in glucose metabolism in the direct subtraction analyses of “treatment responders” vs. “non-responders” (A vs. B and vice versa). (PDF 891 KB) [file 415_2022_11252_MOESM3_ESM.pdf]

PET A (before treatment) vs. healthy controls						
**Area**	**R/L**	**X**	**Y**	**Z**	**T-value**	**Cluster**
**Occipital Pole, Visual cortex V2, BA 18**	**R**	**30**	**-92**	**16**	**6,13**	**539**
Visual cortex V1 BA 17		14	-88	18	5,53	
Visual cortex V3/V4/V2, BA 18		38	-88	14	5,28	
**Visual cortex V1**	**R**	**22**	**-98**	**-18**	**4,31**	**61**
**Lateral occipital cortex, inferior division, Visual cortex V5**	**R**	**52**	**-70**	**-4**	**4,91**	**229**
Visual cortex V4, V3		40	-80	-16	4,50	
**Occipital pole, Visual cortex V2/V1**	**L**	**-12**	**-94**	**12**	**3,78**	**22**
**Lateral occipital cortex, Visual Cortex V2**	**L**	**-30**	**-90**	**14**	**4,56**	**89**
**Lateral occipital cortex, inferior division, Visual cortex V5**	**L**	**-40**	**-72**	**-4**	**5,26**	**414**
Visual cortex V4, V3, V2		-36	-88	-14	4,32	
Visual cortex V4, V3, V2		-36	-88	-8	4,07	
**Inferior parietal lobule, supramarginal gyrus**	**L**	**-58**	**-30**	**38**	**4,91**	**122**
Postcentral gyrus, primary sensory cortex		-54	-22	46	4,19	
**Premotor cortex, precentral gyrus BA 6**	**L**	**-54**	**4**	**34**	**4,52**	**221**
Precentral cortex, corticospinal tract		-44	**-2**	**32**	4,01	
Pre-/postcentral cortex, primary motor, primary somatosensory cortex		-52	-8	40	3,99	
**Postcentral gyrus, secondary somatosenory, OP4**	**L**	**-62**	**-18**	**22**	**3,72**	**16**
**Middle /superior temporal gyrus, posterior division, BA 20/22**	**R**	**64**	**-24**	**-6**	**6,06**	**476**
Middle temporal gyrus, posterior division		60	-16	-24	4,53	
Middle temporal gyrus, posterior division		60	-12	-16	3,89	
**Middle temporal gyrus, anterior division**	**R**	**54**	**6**	**-38**	**5,62**	**89**
**Inferior temporal gyrus, temporooccipital part**	**R**	**56**	**-52**	**-14**	**3,95**	**36**
**Middle / superior temporal gyrus, posterior division, BA 20/22**	**L**	**-64**	**-24**	**-10**	**3,99**	**187**
Superior temporal gyrus		-50	-12	-12	3,91	
**Middle temporal gyrus, posterior division**	**L**	**-60**	**-40**	**-6**	**3,75**	**15**
**Inferior temporal gyrus, posterior division**	**L**	**-54**	**-42**	**-22**	**3,50**	**72**
**Supramarginal/superior temporal gyrus**	**L**	**-58**	**-42**	**12**	**4,04**	**64**
Inferior temporal gyrus, posterior division		-54	-42	-24	3,26	
**Anterior insula cortex**	**L**	**-26**	**16**	**-12**	**3,51**	**59**
**Frontal Pole**	**L**	**-40**	**38**	**-10**	**4,63**	**111**
**Frontal Pole**	**R**	**30**	**44**	**-8**	**3,90**	**50**
**Healthy controls v. PET A (before treatment)**						
**Area**	**R/L**	**X**	**Y**	**Z**	**T-value**	**Cluster**
**Temporal Pole / inferior frontal gyrus**	**R**	**56**	**8**	**-4**	**3,78**	**57**
**Optic radiation / lateral occipital cortex**	**R**	**36**	**-60**	**18**	**4,28**	**60**
**Optic radiation / lateral occipital cortex**	**L**	**-34**	**-56**	**14**	**3,58**	**57**
**Parahippocampal gyrus**	**R**	**14**	**-8**	**-32**	**3,61**	**51**
**Parahippocampal gyrus**	**L**	**-8**	**-12**	**-38**	**4,68**	**59**
**Cerebellum (I-IV, V)**	**R**	**14**	**-44**	**-18**	**3,88**	**38**

Supplementary Table 1

PET B (on treatment) vs. healthy controls						
Area	R/L	X	Y	Z	T-value	Cluster
**Occipital pole, Visual cortex V1/V2, BA 17/18**	**R**	**20**	**-98**	**-16**	**4,27**	**33**
**Occipital pole, Visual cortex V2, BA 18**	**R**	**18**	**-92**	**18**	**6,3**	**719**
Lateral occipital cortex, superior division		38	-88	16	5,72	
Lateral occipital cortex, superior division		16	-86	30	4,70	
**Lateral occipital cortex, Visual cortex V4/V3**	**R**	**40**	**-80**	**-16**	**4,87**	**251**
Lateral occipital cortex, Visual cortex V5		50	-70	-8	4,70	
**Occipital pole, Visual cortex V2, BA18**	**L**	**-28**	**-90**	**16**	**5,33**	**225**
Occipital pole, Visual cortex V2/V1, BA17/18		-12	-94	12	4,04	
**Lateral occipital cortex, Visual cortex V5**	**L**	**-40**	**-72**	**-4**	**5,24**	**520**
Lateral occipital cortex, Visual cortex V3/V4		-40	-86	-14	4,91	
Inferior temporal gyrus, temporooccipital part		-50	-62	-10	3,88	
**Middle temporal gyrus, posterior division**	**R**	**64**	**-20**	**-10**	**5,84**	**408**
Superior/middle temporal gyrus, superior divison		62	-30	2	4,92	
Secondary somatosenory cortex, parietal operculum OP1		62	-22	22	4,23	
**Inferior/middle temporal gyrus,**	**R**	**54**	**-54**	**-8**	**4,11**	**73**
Angular gyrus/inferior parietal lobule		56	-52	16	4,09	
**Middle temporal gyrus, posterior division**	**L**	**-64**	**-24**	**-10**	**5,52**	**580**
Superior temporal gyrus, posterior division		-62	-16	-6	5,38	
Superior/middle temporal gyrus, posterior division, acoustic cortex/radiation		-52	-16	-10	4,79	
**Inferior parietal lobule, supramarginal gyrus**	**L**	**-56**	**-44**	**22**	**4,63**	**102**
Planum temporale, auditory cortex		-48	-34	8	4,02	
**Inferior temporal gyrus, posterior division**	**L**	**-50**	**-38**	**-24**	**6,25**	**186**
**Middle temporal gyrus, posterior divison/ temporooccipital part**	**L**	**-60**	**-42**	**-6**	**4,72**	**47**
**Postcentral gyrus**	**R**	**64**	**-8**	**40**	**4,27**	**33**
Postcentral gyrus, somatosensory cortex, BA 1/2		58	-16	50	3,73	
**Postcentral gyrus, primary somatosensory cortex BA 1/2**	**L**	**-54**	**-18**	**46**	**4,10**	**59**
Postcentral gyrus, somatosensory cortex BA 1/2		-58	-16	36	3,62	
**Postcentral gyrus, somatosensory cortex, parietal operculum OP4/1**	**L**	**-64**	**-16**	**22**	**4,61**	**80**
**Pre-/postcentral gyrus, motor cortex, BA 4**	**L**	**-48**	**-8**	**34**	**3,93**	**43**
**Putamen**	**L**	**-22**	**20**	**-6**	**3,87**	**14**
**Frontal Pole**	**L**	**-40**	**38**	**-10**	**4,78**	**77**

Healthy controls vs. PET B (on treatment)						
Area	R/L	X	Y	Z	T-value	Cluster
**Cerebellum (Vermis, VI)**	**R**	**4**	**-66**	**-22**	**5,16**	**887**
Cerebellum (I-IV, V)		12	-44	-16	5,16	
Cerebellum (I-IV, V)		8	-52	-12	4,58	
**Cerebellum (VIIb/a)**	**R**	**42**	**-52**	**-58**	**3,86**	**12**
**Cerebellum (VI/V)**	**L**	**-24**	**-50**	**-30**	**3,79**	**14**
**Cerebellum**	**R**	**16**	**-50**	**-38**	**3,76**	**16**
**(Pontine brainstem)**	**L**	**-10**	**-12**	**-34**	**4,73**	**98**
**Parahippocampal gyrus**	**R**	**14**	**-8**	**-32**	**4,27**	**52**
**Thalamus**	**R**	**8**	**-4**	**4**	**3,93**	**12**
**Optic radiation/callosal body/middle temp. gyr.**	**L**	**-30**	**-48**	**26**	**3,92**	**66**
**Middle temporal gyrus/ inferior parietal lobule**	**R**	**42**	**-56**	**10**	**4,93**	**103**

Supplementary Table 2

PET B vs. PET A (on vs. off treatment), 0.001

Area	R/L	X	Y	Z	T-value	Cluster
Supramarginal gyrus	L	-60	-46	48	4,92	10

PET B vs. PET A (on vs. off treatment), 0.005

Area	*R/L*	*X*	*Y*	*Z*	*T-value*	*Cluster*
Supramarginal gyrus	L	-60	-46	48	4,92	29
Superior temporal gyrus / Secondary somatosenory cortex (OP4)	L	-66	-16	2	3,27	112
Middle temporal gyrus / Supramarginal gyrus	R	48	-46	6	3,32	18
Anterior intraparietal sulcus	L	-30	-36	38	4,05	14
Superior parietal lobule 7	L	-36	-60	56	3,96	11
Superior parietal lobule 7	R	10	-62	62	3,96	21
Superior parietal lobule 7	R	8	-48	76	3,39	14
Frontal pole /superior frontal gyrus	L	-22	44	46	3,84	62
Superior frontal gyrus / Premotor area, BA 6	R	24	0	46	3,71	32
Premotor cortex, BA 6	L	-6	-18	44	3,66	111
Juxtapositional Lobule /Premotor cortex, BA 6	L	-4	-4	58	3,57	
Premotor cortex, BA 6	R	12	-2	78	4,3	36
Middle frontal gyrus	R	36	34	16	3,66	12
Optic radiation /lateral geniculate body	R	26	-24	-4	3,58	23
Precuneus Cortex	R	0	-60	18	3,53	36
Visual Cortex (V1, BA17)	R	8	-62	10	3,34	34
Visual cortex (V1, BA17)/optic radiation	R	30	-62	6	4,09	51
Cerebellum VI / Nodule	R	16	-62	-30	3,48	19
Callosal body	L	-6	-36	14	3,58	13

PET A vs. PET B (off vs. on treatment), 0.001

Area	R/L	X	Y	Z	T-value	Cluster
Cerebellum /Crus I	L	-40	-70	-24	6,19	80
Temporal fusiform/parahippocampal gyrus	R	34	-14	-32	5,52	49
Superior parietal lobule/post/precentral gyrus	R	6	-36	56	5,39	14

PET A vs. PET B (off vs. on treatment), 0.005

Area	*R/L*	*X*	*Y*	*Z*	*T-value*	*Cluster*
Cerebellum /Crus I	L	-40	-70	-24	6,19	178
Cerebellum	L	-10	-62	-32	3,43	12
Cerebellum/Lingual gyrus (VI, Vermis)	R	6	68	-12	3,50	17
Cerebellum (VI, Crus I)	R	34	-62	-26	3,44	15
Posterior insular cortex	L	-36	-16	8	3,68	12
Posterior insular cortex	R	40	-16	12	3,62	10
Temporal fusiform/parahippocampal gyrus	R	34	-14	-32	5,52	97
Temporal fusiform/ parahippocampal gyrus	R	26	-36	-24	3,53	14
Temporal fusifom cortex/parahippocampal gyrus/optic radiation	R	30	-40	-10	3,47	17
Middle temporal gyrus	R	74	-36	-14	4,99	23
Middle/superior temporal gyrus	R	56	-32	0	3,42	18
Middle/superior temporal gyrus	R	58	-36	10	3,15	11
Superior parietal lobule/post/precentral gyrus	R	6	-36	56	5,39	43
Juxtapositional lobule/premotor cortex, BA 6	R	6	0	62	4,20	39
Lateral occipital cortex	L	-46	-72	20	3,39	23
Visual cortex /occipital pole V2/3	R	38	-94	-6	3,61	15

Supplementary Table 3

PET A Responders (before treatment) vs. healthy controls						
Area	R/L	X	Y	Z	T-value	Cluster
**Visual cortex V2, V1, BA 18/17**	**R**	**32**	**-94**	**16**	**7,69**	**136**
Visual cortex V2, V1, BA 17/18		14	-88	18	4,28	
**Visual cortex V2, V3, BA 18**	**L**	**-30**	**-990**	**12**	**4,2**	**16**
**Lateral occipital cortex, Visual cortex V5**	**R**	**54**	**-70**	**-4**	**5,37**	**27**
**Lateral occipital cortex, Visual cortex V5**	**L**	**-38**	**-72**	**-2**	**5,24**	**76**
**Lateral occipital cortex / inferior parietal lobule**	**L**	**-42**	**-82**	**30**	**4,66**	**13**
**Middle temporal gyrus, posterior division, BA 21**	**L**	**-60**	**-38**	**-6**	**6,52**	**74**
**Middle temporal gyrus, posterior division**	**L**	**-60**	**-22**	**-18**	**4,23**	**27**
**Planum temporale/superior temporal gyrus**	**R**	**62**	**-26**	**12**	**4,77**	**127**
Middle temporal gyrus, posterior division		54	-24	-6	4,34	
Planum temporale/superior temporal gyrus		54	-18	-2	4,22	
**Superior temporal gyrus**	**L**	**-58**	**-38**	**10**	**6,01**	**75**
**Temporal pole, middle temporal gyrus**	**R**	**54**	**6**	**-38**	**4,91**	**15**
**Precentral gyrus, primary motor, BA 4**	**L**	**-44**	**-4**	**32**	**4,99**	**196**
Precentral gyrus, premotor cortex, BA 6		-52	0	34	4,90	
**Postcentral gyrus / supramarginal gyrus**	**L**	**-54**	**-26**	**40**	**4,38**	**23**
**Putamen/Pallidum**	**L**	**-28**	**10**	**4**	**6,19**	**356**
**Putamen**	**R**	**28**	**10**	**-4**	**4,9**	**39**
**Parahippocampal gyrus, posterior division**	**L**	**-22**	**-40**	**-10**	**4,75**	**24**
**Frontal pole**	**R**	**30**	**56**	**34**	**4,88**	**28**
**Frontal pole**	**L**	**-40**	**40**	**-8**	**4,63**	**62**
**Middle frontal gyrus**	**L**	**-38**	**20**	**38**	**4,58**	**20**

Healthy controls v. PET A Responders (before treatment)						
Area	R/L	X	Y	Z	T-value	Cluster
**Cerebellum, anterior lobe/culmen, I-IV**	**R**	**4**	**-54**	**-10**	**8,46**	**1145**
Cerebellum, anterior lobe/culmen, I-IV		2	-52	-16	6,97	
Cerebellum, anterior lobe/culmen, V, I-IV		16	-44	-16	5,18	
**Cerebellum Crus II, VIIb**	**R**	**14**	**-78**	**-46**	**4,97**	**130**
**Cerebellum VIIb, a, Crus II**	**L**	**-8**	**-72**	**-50**	**4,56**	**50**
**Cerebellar Crus II,I**	**R**	**22**	**-90**	**-30**	**4,54**	**21**
**Cerebellar Crus I,II**	**L**	**-30**	**-80**	**-32**	**4,42**	**44**
**Cerebellum Crus VI**	**R**	**26**	**-68**	**-28**	**4,39**	**76**
Cerebellum Crus I		42	-56	-32	3,98	
**Callosal body, cingulate posterior division**	**L**	**-6**	**-44**	**18**	**6,00**	**99**
**Optic radiation, callosal body**	**L**	**-38**	**-52**	**-8**	**4,85**	**10**
**Hippocampus**	**L**	**-34**	**-34**	**-10**	**4,52**	**11**
**Inferior parietal lobule**	**R**	**42**	**-58**	**18**	**4,61**	**51**

Supplementary Table 4

PET B Responders (on treatment) vs. healthy controls, 0.001						
**Area**	**R/L**	**X**	**Y**	**Z**	**T-value**	**Cluster**
**Occipital pole, Visual cortex V2/V1, BA 18/17**	**R**	**16**	**-92**	**18**	**6,46**	**576**
Lateral occipital cortex, superior division		38	-88	16	5,51	
Occipital pole, Visual cortex V2/V1, BA 18/17		30	-94	14	5,51	
**Occipital pole**	**R**	**20**	**-98**	**-20**	**4,51**	**30**
Occipital pole		20	-104	-10	3,89	
**Lateral occipital cortex, inferior division**	**R**	**40**	**-82**	**-16**	**4,66**	**58**
**Lateral occipital cortex, Visual cortex V4/3**	**R**	**52**	**-74**	**-4**	**4,62**	**52**
**Occipital pole, Visual cortex V2, BA 18**	**L**	**-28**	**-90**	**16**	**5,44**	**703**
Lateral occipital cortex, Visual cortex V4/3		-40	-86	-10	5,42	
Occipital pole, Visual cortex V1/2, BA 17/18		-10	-92	12	4,52	
**Precuneus /superior parietal lobule**	**L**	**-12**	**-48**	**42**	**5,15**	**20**
**Middle temporal gyrus, posterior division**	**R**	**64**	**-20**	**-10**	**5,21**	**314**
Inferior temporal gyrus, posterior division		58	-18	-26	4,77	
Middle/inferior temporal gyrus, posterior division		60	-28	-16	4,57	
**Inferior temporal gyrus, temporooccipital part**	**R**	**58**	**-52**	**-14**	**3,95**	**22**
**Middle temporal gyrus, temporooccipital part**	**L**	**-58**	**-46**	**-6**	**6,11**	**85**
**Inferior temporal gyrus, posterior division**	**L**	**-50**	**-38**	**-24**	**5,05**	**84**
**Middle/superior temporal gyrus, posterior d.**	**L**	**-64**	**-22**	**-10**	**7,11**	**679**
Superior temp. gyrus, somatosensory cortex, OP 1		-60	-26	6	4,98	
Supramarginal gyrus/Inferior parietal lobue		-58	-44	22	4,7	
**Post-/precentral gyrus**	**R**	**64**	**-8**	**40**	**4,28**	**14**
**Postcentral gyrus, primary somatosensory, BA 1/2**	**R**	**58**	**-18**	**50**	**4,06**	**19**
**Postcentral gyrus, primary somatosensory, BA 1**	**L**	**-54**	**-14**	**54**	**4,2**	**34**
Postcentral gyrus, primary somatosensory, BA 1/2)		-54	-18	44	3,91	
**Pre-/postcentral gyrus, primary motor, BA 4; somatosensory, BA 3)**	**L**	**-46**	**-8**	**34**	**4,24**	**43**
**Precentral gyrus, premotor /primary motor, BA 6/4**		**-52**	**-2**	**36**	**3,7**	

Healthy controls vs. PET B Responders (on treatment)						
**Area**	**R/L**	**X**	**Y**	**Z**	**T-value**	**Cluster**
**Cerebellar vermis (VIIIa/b, VI; hemisphere VI)**	**L**	**-2**	**-62**	**-20**	**9,29**	**1835**
Cerebellum I-V	R	14	-46	-12	8,3	
Cerebellum I-V, V	R	6	-54	-12	8,22	
**Cerebellum, Crus I, VI**	**R**	**42**	**-46**	**-36**	**4,37**	**30**
**Middle cerebellar peduncle, VI/V**	**L**	**-22**	**-48**	**-30**	**4,74**	**70**
**Juxtapositional Lobule, supplementary motor cortex/premotor cortex, BA 6**	**R**	**6**	**8**	**60**	**5,18**	**137**
**Middle temporal gyrus/inferior parietal lobule**	**R**	**42**	**-56**	**10**	**4,79**	**70**
**Intraparietal sulcus/callosal body**	**L**	**-30**	**-50**	**28**	**5,08**	**116**

Healthy controls vs. PET B Responders (on treatment), 0.05 corrected						
**Area**	**R/L**	**X**	**Y**	**Z**	**T-value**	**Cluster**
**Cerebellar vermis (VIIIa/b, VI; hemisphere VI)**	**L**	**-2**	**-62**	**-20**	**9,29**	**91**
Cerebellum I-V, V	R	6	-54	-12	8,22	

Supplementary Table 5

Responders PET B vs. PET A (on vs. off treatment), 0.001

Area	R/L	X	Y	Z	T-value	Cluster
**Post-/Precentral gyrus, somatosensory, BA 1/premotor BA 6)**	**R**	**66**	**-6**	**30**	**8,71**	**17**
**Lateral occipital cortex /optic radiation**	**R**	**32**	**-62**	**18**	**6,24**	**20**
**Juxtapositional lobule cortex, supplementary motor/premotor cortex, BA 6**	**L**	**-6**	**-8**	**52**	**6,03**	**27**
Juxtapositional lobule cortex, supplementary motor/premotor cortex, BA 6		-8	-14	46	4,82	
**Inferior temporal gyrus, posterior division**	**L**	**-52**	**-28**	**-26**	**5,77**	**12**
**Juxtapositional lobule cortex, supplementary motor**	**L**	**-6**	**6**	**56**	**5,16**	**16**

Responders PET B vs. PET A (on vs. off treatment), 0.005

Area	*R/L*	*X*	*Y*	*Z*	*T-value*	*Cluster*
Postcentral gyrus, somatosensory, BA 1/3	***R***	***66***	***-6***	***30***	***8,71***	***39***
Postcentral gyrus, somatosensory, BA 1/ inferior parietal lobule, BA 40	***R***	***44***	***-36***	***62***	***4,97***	***30***
Postcentral gyrus, somatosensory, BA 3	***L***	***-18***	***-34***	***60***	***5,39***	***10***
Juxtapositional lobule cortex, supplementary motor / premotor cortex, BA 6	***L***	***-6***	***-8***	***52***	***6,03***	***166***
Juxtapositional lobule cortex, supplementary motor / premotor cortex, BA 6		*-6*	*6*	*56*	*5,16*	
Juxtapositional lobule cortex, supplementary motor / premotor cortex, BA 6		*-8*	*-14*	*46*	*4,82*	
Precentral gyrus / Premotor cortex BA 6	***L***	***-44***	***-12***	***60***	***3,76***	***14***
Intracalcarine cortex /Visual cortex V1, BA 17	***R***	***14***	***-72***	***12***	***4,81***	***136***
Intracalcarine cortex / Visual cortex V1/V2		*18*	*-62*	*4*	*4,62*	
Optic radiation / occipital white matter / callosal body	***R***	***32***	***-62***	***18***	***6,24***	***72***
Cuneal cortex / Visual cortex V2	***R***	***12***	***-84***	***32***	***4,26***	***18***
Angular gyrus / lateral occipital cortex, superior division / inferior parietal lobule	***L***	***-46***	***-58***	***32***	***3,8***	***32***
Lateral occipital cortex, superior division / superior parietal lobule /precuneus, BA 7	***L***	***-20***	***-64***	***58***	***5,92***	***44***
Lateral occipital cortex, superior division / inferior parietal lobule	***L***	***-24***	***-82***	***40***	***4,67***	***20***
Lateral occipital cortex, superior division	***R***	***34***	***-58***	***38***	***4,43***	***13***
Precuneus	***R***	***12***	***-50***	***36***	***4,42***	***15***
Precuneus	***R***	***0***	***-54***	***14***	***4,21***	***23***
Supramarginal/superior temporal gyrus	***R***	***46***	***-36***	***10***	***5,22***	***28***
Inferior temporal gyrus, temporooccipital part	***R***	***52***	***-56***	***-22***	***5,13***	***10***
Inferior temporal gyrus, posterior division	***L***	***-52***	***-28***	***-26***	***5,77***	***36***
Middle/inferior frontal cortex	***R***	***38***	***34***	***16***	***4,57***	***27***

Responders PET A vs. PET B (off vs. on treatment), 0.001

Area	R/L	X	Y	Z	T-value	Cluster
Midline cerebellum / VIIIa	L	-10	-62	-34	6,01	15
Posterior insular cortex	L	-38	-18	6	5,98	11
Juxtapositional lobule cortex, supplementary motor/premotor area, BA 6	R	6	4	62	5,25	13
Frontal white matter /anterior callosal body	L	-22	36	14	5,04	11

Responders PET A vs. PET B (off vs. on treatment), 0.005

Area	*R/L*	*X*	*Y*	*Z*	*T-value*	*Cluster*
Midline cerebellum /VIIIa	***L***	***-10***	***-62***	***-34***	***6,01***	***93***
Vermis, VIIIa, VIIb, Crus II		*-4*	*-70*	*-38*	*4,7*	
Cerebellum, Crus I/VI	***L***	***-38***	***-68***	***-24***	***4,38***	***31***

Midbrain/Pons	***L***	***-10***	***-18***	***-18***	***5,01***	***53***
(Para)hippocampal gyrus /temporal fusiform gyrus	***R***	***34***	***-14***	***-28***	***4,08***	***54***
Temporal pole /parahippocampal gyrus	***R***	***22***	***4***	***-40***	***3,79***	***11***
(Para-)hippocampal gyrus /amygdala	***L***	***-24***	***-2***	***-20***	***4,48***	***17***
Posterior insular cortex	***L***	***-38***	***-18***	***6***	***5,98***	***31***
Occipital fusifrom / lingual gyrus, visual cortex V3/4	***L***	***-26***	***-66***	***-6***	***5,31***	***27***
Lateral occipital cortex /precuneus / superior parietal lobule, BA 7	***L***	***-10***	***-78***	***56***	***5,25***	***16***
Juxtapositional lobule, supplementary motor/premotor area, BA 6	***R***	***6***	***4***	***62***	***5,25***	***39***
Callosal body / frontal white matter	***L***	***-22***	***36***	***14***	***5,04***	***59***
callosal body / frontal white matter	***L***	***-20***	***16***	***32***	***3,63***	***28***
Callosal body	***R***	***22***	***34***	***4***	***4,06***	***52***

Supplementary Table 6
